# Serotonin, melatonin and their precursors and metabolites and vitamin D_3_ derivatives in honey

**DOI:** 10.32794/mr112500137

**Published:** 2022-09-30

**Authors:** Tae-Kang Kim, Adrian Fabisiak, Pawel Brzeminski, Russel J. Reiter, Andrzej T. Slominski

**Affiliations:** 1 Department of Dermatology, The University of Alabama at Birmingham, Birmingham, Alabama 35294, USA; 2 Department of Chemistry, University of Warsaw, 02-093 Warsaw, Poland; 3 Department of Cell Systems and Anatomy, UT Health, Long School of Medicine, San Antonio, Texas 78229, USA; 4 Pathology and Laboratory Medicine Service, VA Medical Center, Birmingham, Alabama 35294, USA

**Keywords:** melatonin, serotonin, honey, bees, AMK, AFMK, biogenic amines, secosteroids

## Abstract

We are commenting recent discoveries on the presence of L-DOPA, dopamine, 5-hydroxytryptophan, tryptamine, serotonin, *N*-acetylserotonin, melatonin, 2-hydroxymelatonin, AFMK and AMK in honey. Serotonin and melatonin, products of the tryptophan metabolism, are widely produced in nature, serving as hormones, neurotransmitters, biological regulators, neurotransmitters and antioxidants, in a context dependent fashion. Dopamine and tryptamine are important neurotransmitters across different species. Honey is used as one of the most popular healthy food substances. Detection of above molecules in honey accompanied by detection of vitamin D_3_ and its hydroxyderivatives, is consistent with their detection in insects and plants. Their presence in honey enhances spectrum of its beneficial effects for human health and implicates that these molecules must play important role in social insects physiology, bees development and colony functions.

## 1. INTRODUCTION

Honey is produced by honey bees (mainly) and some other social insects from the nectar or honeydew collected from living plants (1). It is one of the most popular food substances with sweet taste and healthy ingredients such as polyphenols and flavonoids. Eva Crane suggested that honey was used by human at for least 8,000 years ago (2). Also, Ayurveda suggested the use of honey for a variety of diseases. Honey is used as a good nutrition source since it contains high sugars including fructose and glucose, as well as in traditional medicine from ancient time for treatment for minor wounds and burns and other pathologies (3–5). In addition, honey has antioxidant (6), anti-inflammatory (7) antibiotics (8, 9), cough suppressing (10) and anticancer (11) properties. However, there is shortage of information on the ingredients, which could affect these biological functions.

**Scheme 1.**
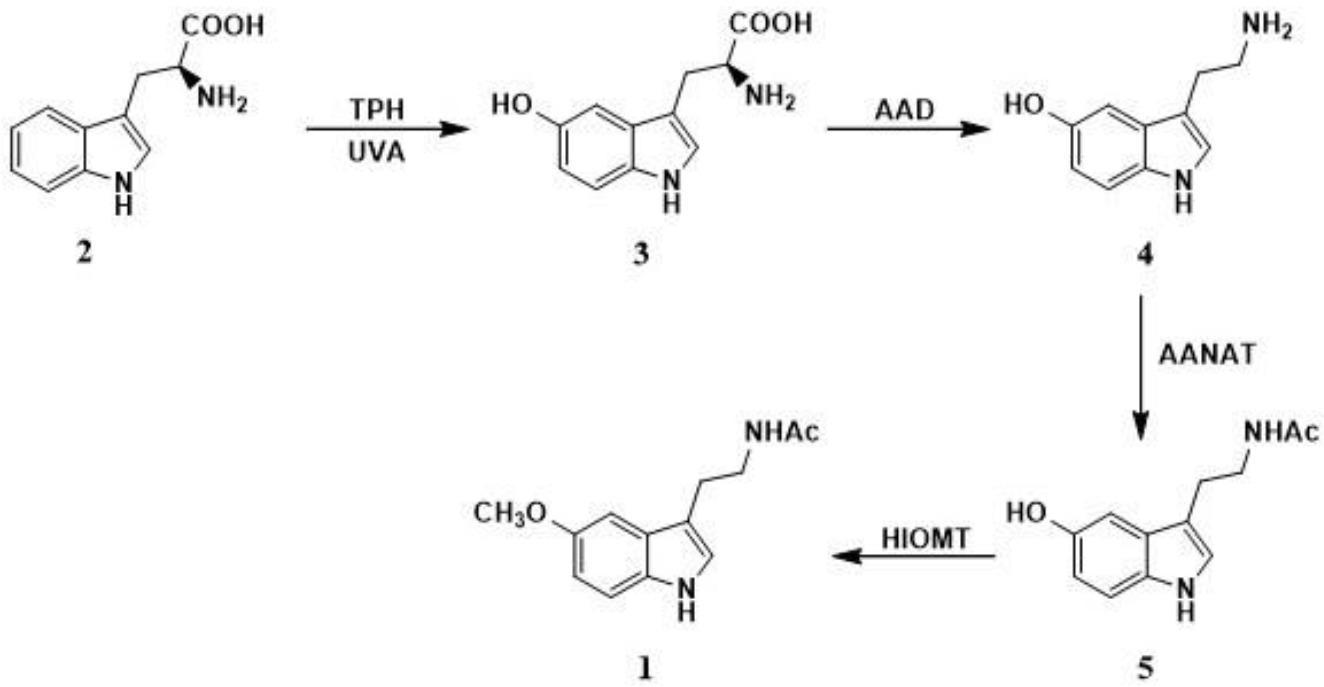
Melatonin biosynthesis.

Melatonin (Scheme 1) (**1**) and serotonin are synthesized from *L*-tryptophan (**2**) through several enzymatic reactions within its aromatic benzene ring and aliphatic chain. First, *L*-tryptophan is hydroxylated by tryptophan hydroxylase (TPH) or through non-enzymatic reaction induced by ultraviolet A (UVA) forming 5-hydroxytryptophan (**3**) which is further decarboxylated in the presence of aromatic amino acid decarboxylase (ADD) to 5-hydroxytryptamine (**4**) most widely known as serotonin. Next, serotonin undergoes acetylation reaction on its primary amine group to produce *N*-acetylserotonin (**5**) by arylalkylamine-*N*-acetyltransferase (AANAT) or arylamine *N-*acetyltransferase (NAT). The final biosynthetic step in animals is performed in the presence of hydroxyindole-*O*-methyltransferase (HIOMT) resulting in the formation of melatonin as a methyl ether derivative of *N*-acetylserotonin (Scheme 1).

**Scheme 2.**
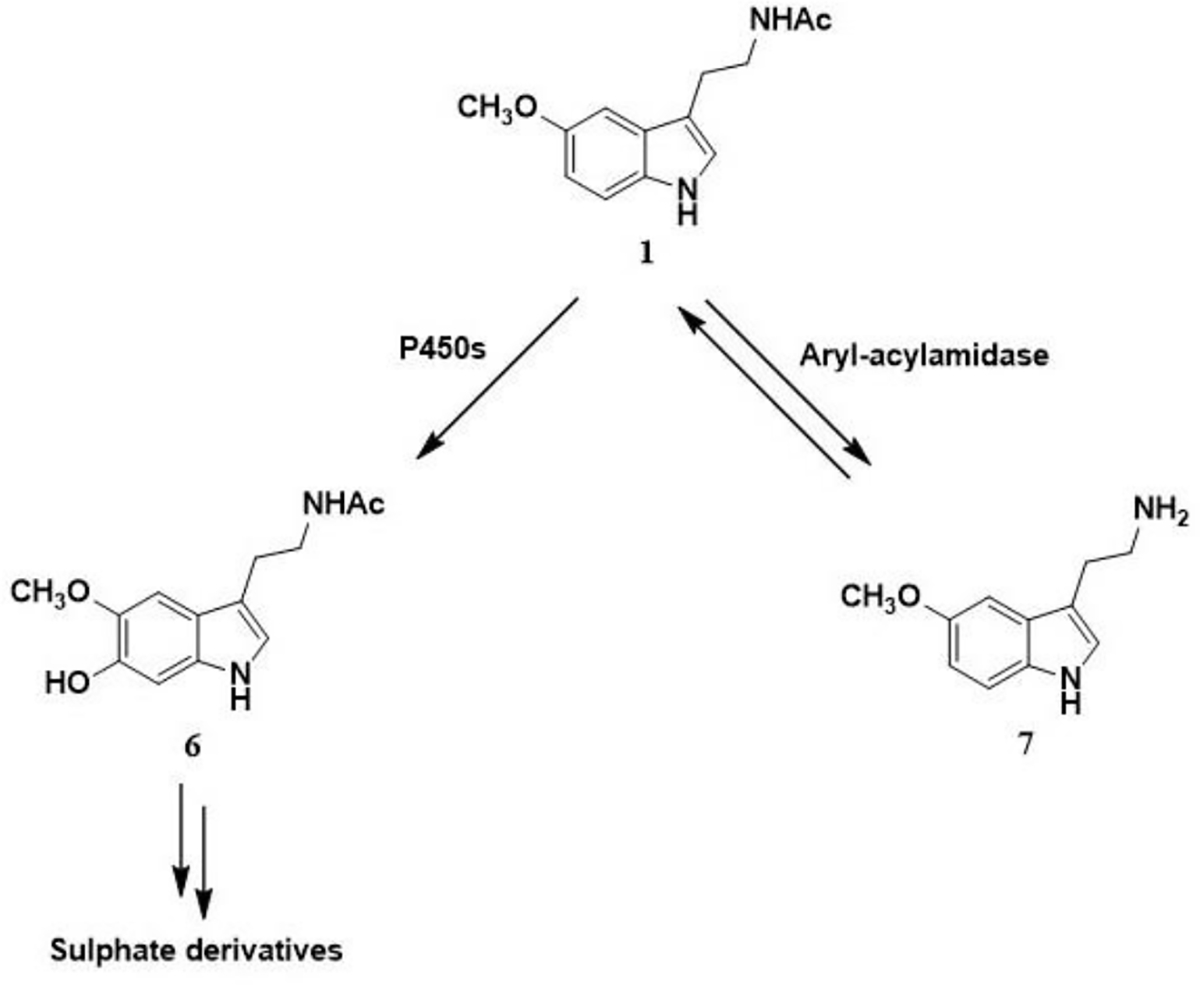
The indolic pathway

**Scheme 3.**
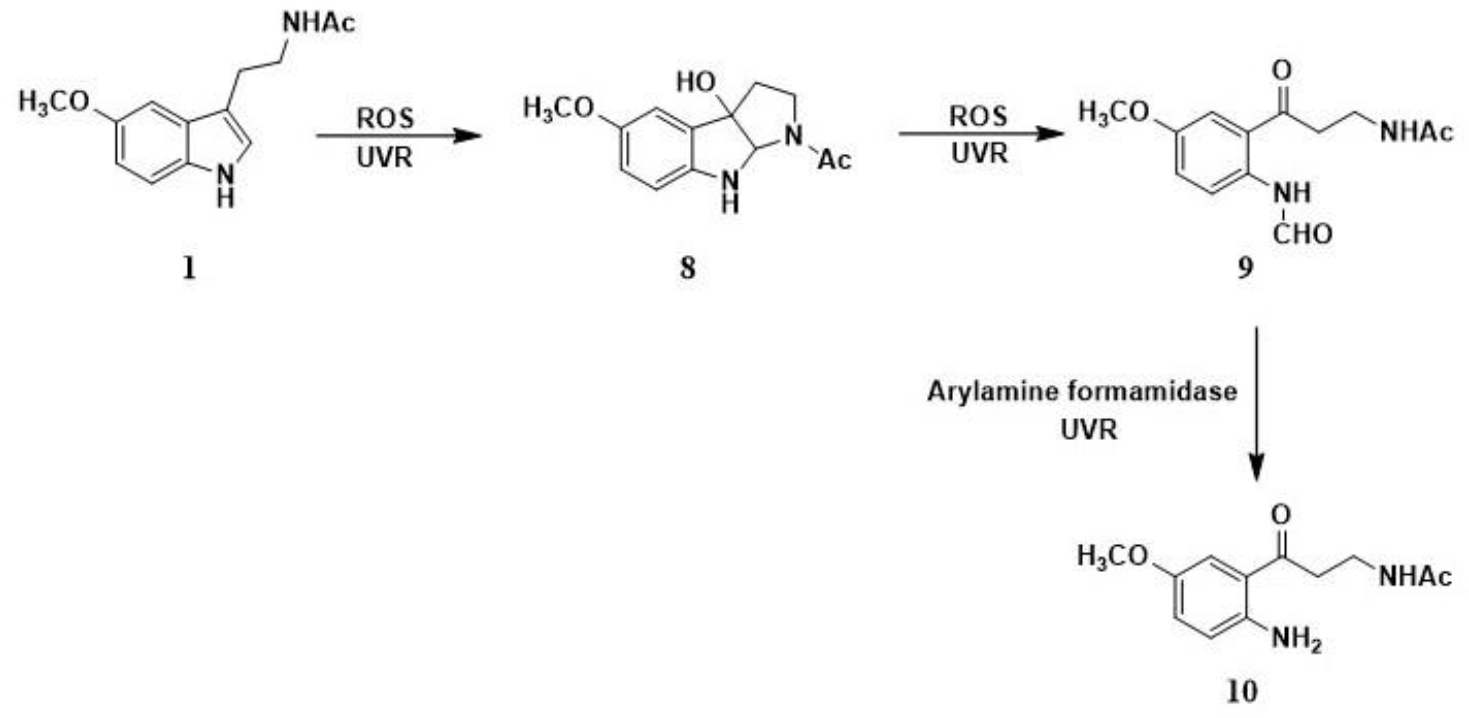
The kynurenic pathway

Melatonin is metabolized enzymatically through the indolic (Scheme 2) and both non-enzymatically and enzymatically through kynurenic (Scheme 3) pathway. The indolic pathway produces 6-hydroxymelatonin (**6**) which is further conjugated with sulphates in the liver by cytochrome P_450_ monooxygenases. Moreover, 5-methoxytryptamine (**7**) is also produced as a result of deacetylation reaction carried out by melatonin-deacetylating enzymes. On the other hand, melatonin also undergoes numerous radical reactions in the kynurenic pathway mediated by reactive oxygen species (ROS) or ultraviolet radiation (UVR). These scavenging cascade reactions go through the formation of cyclic 3-hydroxymelatonin (**8**) to produce AFMK (*N*^1^-acetyl-*N*^2^-formyl-5-methoxykynuramine, **9**) and AMK (*N*^1^-acetyl-5-methoxykynuramine, **10**) as final metabolites while confirming the antioxidant properties of melatonin.

The sensitivity of analytical instruments has recently been dramatically improved resulting in the ability to detect small amounts of biologically active molecules in many food sources. To further investigate beneficial properties of honey, we evaluated the presence of serotonin, melatonin and their metabolites, as well as vitamin D_3_ derivatives in honey using the Xevo G2-XS qToF LC-Ms system (12, 13). Others have used HPLC with diode array detector to evaluate the biogenic amines and their precursors and melatonin content (14).

## 2. INGREDIENTS OF HONEY

Honey contains monosaccharide sugars such as glucose and fructose, many biochemical substances including enzymes, minerals, vitamins, amino acids, being good nutrient sources as well as it has medicinally healthy ingredients such as polyphenols and flavonoids acting as antioxidants. The composition of them depends on floral and geographical origin (15, 16). Chemically complex polyphenols (17) are produced by plants, being present in fruits, vegetables and nuts (18) and have antimicrobial and anticancer properties (19, 20). Both, polyphenols and flavonoids are transferred to honey by bees.

Tryptophan, a precursor to serotonin melatonin, is synthesized in plants and decarboxylated to tryptamine by tryptophan decarboxylase followed by converting to serotonin by tryptamine 5-hydroxylase (21–23). In plants, melatonin is synthesized from serotonin and from 5-methoxytryptamine which is a metabolite of serotonin (24). Serotonin synthesis from 5-hydroxytryptophan, characteristic for animal kingdom, can also occur in plants, however, to a lesser degree (21).

**Fig. 1.**
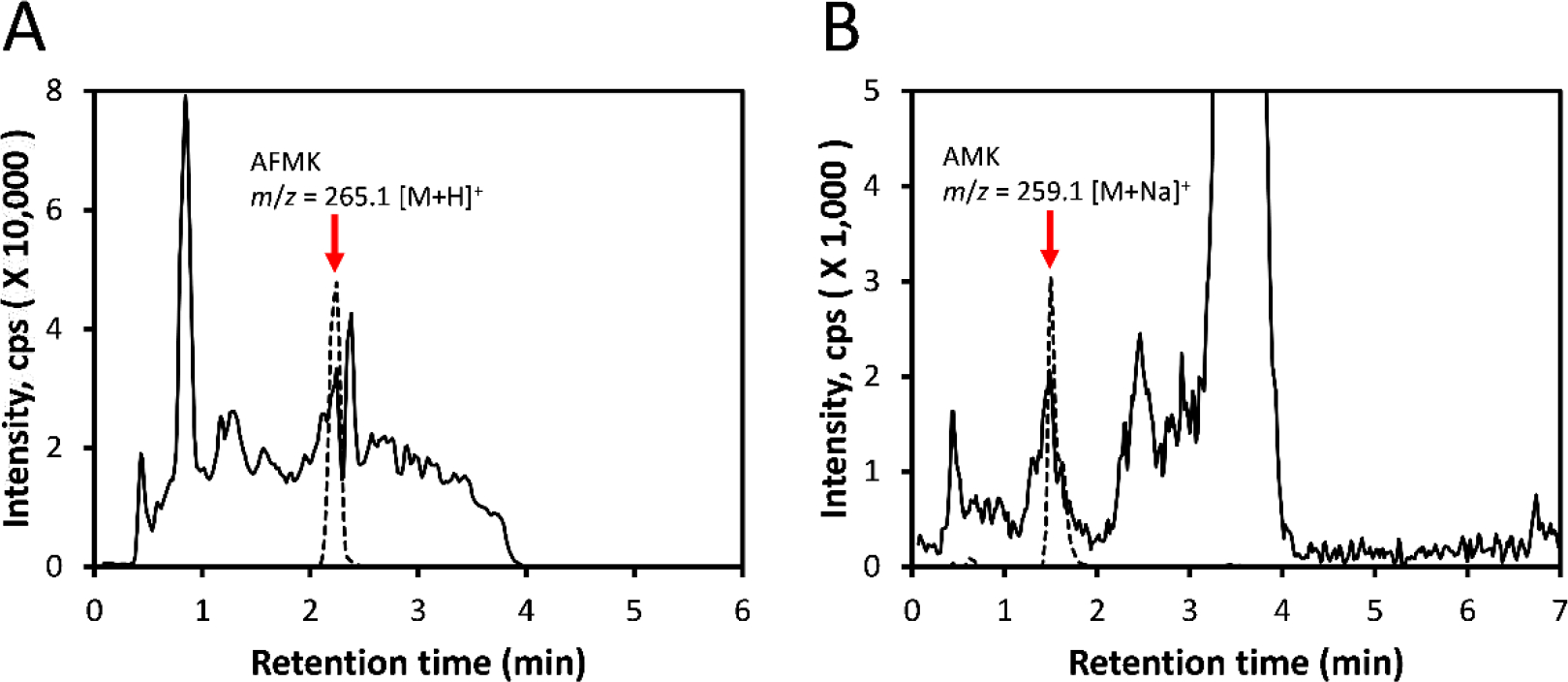
The chromatography of AFMK and AMK A. Detection of AFMK in Australian honey. B. Detection of AMK in US commercial honey. The honey was extracted with methylene chloride previously described in our reports (12). LC-MS detection was performed using Xevo G2 XS qTof LC–MS system (Waters, Milford, MA) equipped with a Zorbax Eclipse Plus C18 column (2.1 × 50 mm, 1.8 μm) (Agilent Technology, Santa Clara, CA, USA). The elution and mass scanning conditions were the same as in the previous report (12). Extracted ion chromatograms (EICs) are shown with m/z = 265.1 [M+H]^+^ for AFMK and m/z = 259.1 [M+Na]^+^ for AMK by using Waters MassLynx 4.1 software. The broken line shows the overlay of the corresponding standards: AFMK [Intensity, cps (X 300.000)] and AMK [Intensity, cps (X 150,000)].

Recently, we detected tryptophan, serotonin, *N*-acetylserotonin (NAS), melatonin and its metabolites such as 2-hydroxymelatonin in honey using the q-ToF LC-Ms system (12). Their detection and the contents were dependent on the source of honey and extraction methods. We are supplementing these studies by additional detection of melatonin kynurenic metabolites in the honey including AFMK and AMK for the first time (Figure. 1).

The most recent study from Brazil, has not only confirmed the detection of tryptophan, serotonin, NAS and melatonin in Brazilian honey, but also reported presence of 5-hydroxytryptophan, tryptamine, dopamine and its precursor L-DOPA (14). Their detection and concentrations were dependent on seasons, year, flowering, and climate (14). In this study, using HPLC they could detect higher amounts of serotonin and melatonin comparing to our results, which can be explained by different extraction methods and source of honey. Their additional detection of L-DOPA, dopamine and tryptamine are significant since two latter molecules can serve as neurotransmitters across different species. In addition, L-DOPA, while serving as a precursor to catecholamines and melanin, can also act as a hormone-like molecule (25, 26). The acidic pH of the honey would prevent L-DOPA and dopamine autoxidation (27). Both studies are important, because presence of biogenic amines and melatonin and precursors and metabolites indicate their contribution to nutritional, health, and perhaps addictive properties of the honey. This should be a subject of further investigations. Importantly, bees do not produce biogenic amines and melatonin for human and some animal consumption, but for their own use. Therefore, this production must a part of physiological processes affecting bees’ development, behavior, well-being and functioning of the colony. These can be regulated by environmental factors, as can be deduced from the variability of their contentment reported by us (12) and Borges *et al*. (14). Additional detection, of AFMK and AMK shown in Figure 1, extends spectrum of melatonin-derived molecules with anti-oxidative properties further contributing to beneficial properties of honey.

As relates to presence of other small bioregulatory molecules in the honey, we also reported presence of 7-dehydrocholesterol (7DHC), vitamin D3 (D_3_) and of 20S(OH)7DHC, 20S(OH)D_3_, 1α,20S(OH)_2_D_3_, 25(OH)D_3_ and 1α,25(OH)_2_D_3_, implying their production by the bees (13). The precise biochemistry and photochemistry of these processes and source of precursor molecules remain to be defined (13). This increase spectrum of beneficial ingredients presented in honey.

## 3. CONCLUSIONS

Recent studies have extended the spectrum of beneficial molecules present in the honey, to biogenic amines with their precursors, melatonin with their metabolites and secosteroidal molecules. They indicate that these molecules are produced during bees’ life cycle, with production changing in response to environmental changes and stressful stimuli. These molecules would not only contribute to social interactions, bees development, colony functions and health of individual bees, but also pass some of these beneficial functions to humans and animals after honey digestion.
